# Novel Control by the CA3 Region of the Hippocampus on Neurogenesis in the Dentate Gyrus of the Adult Rat

**DOI:** 10.1371/journal.pone.0017562

**Published:** 2011-03-18

**Authors:** Jian Xin Liu, Scarlett B. Pinnock, Joe Herbert

**Affiliations:** 1 Institute of Neurobiology, School of Medicine of Xi'an Jiaotong University, Xi'an, P.R.China; 2 Cambridge Centre for Brain Repair, Department of Clinical Neurosciences, and Department of Physiology, Development and Neuroscience, University of Cambridge, Cambridge, United Kingdom; Tokyo Medical and Dental University, Japan

## Abstract

The dentate gyrus is a site of continued neurogenesis in the adult brain. The CA3 region of the hippocampus is the major projection area from the dentate gyrus. CA3 sends reciprocal projections back to the dentate gyrus. Does this imply that CA3 exerts some control over neurogenesis? We studied the effects of lesions of CA3 on neurogenesis in the dentate gyrus, and on the ability of fluoxetine to stimulate mitotic activity in the progenitor cells. Unilateral ibotenic-acid generated lesions were made in CA3. Four days later there was no change on the number of either BrdU or Ki67-positive progenitor cells in the dentate gyrus. However, after 15 or 28 days, there was a marked reduction in surviving BrdU-labelled cells on the lesioned side (but no change in Ki-67+ cells). pCREB or Wnt3a did not co-localise with Ki-67 but with NeuN, a marker of mature neurons. Lesions had no effect on the basal expression of either pCREB or Wnt3a. Subcutaneous fluoxetine (10 mg/kg/day) for 14 days increased the number of Ki67+ cells as expected on the control (non-lesioned) side but not on that with a CA3 lesion. Nevertheless, the expected increase in BDNF, pCREB and Wnt3a still occurred on the lesioned side following fluoxetine treatment. Fluoxetine has been reported to decrease the number of “mature” calbindin-positive cells in the dentate gyrus; we found this still occurred on the side of a CA3 lesion. We then showed that the expression GAP-43 was reduced in the dentate gyrus on the lesioned side, confirming the existence of a synaptic connection between CA3 and the dentate gyrus. These results show that CA3 has a hitherto unsuspected role in regulating neurogenesis in the dentate gyrus of the adult rat.

## Introduction

A striking feature of adult neurogenesis in the dentate gyrus of the hippocampus is the number of factors that can alter its' rate. The list includes: exercise, learning, stress, compounds that alter nitric oxide, excess adrenocortical glucorticoids, and drugs that regulate serotonin, such as the SSRI (selective serotonin reuptake inhibitor) fluoxetine.[1], [2], [3], [4], [5], [6] This list is a mixture of external agents (such as exercise, or stress), internal responses (such as glucocorticoids) and potential neural mechanisms (such as serotonin), and it is not always clear how these relate to one another, though there is evidence of interactions between them: for example, glucocorticoids and serotonin [4]. Neither is the site of action of these agents really understood. It has been assumed that they act within the gyrus, since this is the site of the progenitor cells, as well as the surrounding milieu which is presumed, in some way, to provide a suitable environment not only for neurogenesis itself, but also for allowing connections of newly formed cells to the appropriate afferent and efferent sites[7], [8].

CA3 is the major efferent site for the neurons of the dentate gyrus[9]. It is known that both newly-formed cells, as well as dentate-derived grafts from neonatal animals, send projections to CA3 [10]. What is not known is whether CA3 exerts any reciprocal influence on neurogenesis in the dentate gyrus. Yet there are clues that it might: for example, lesions of CA3 result in excess sprouting of mossy fibres within the dentate gyrus [11], [12], [13], and there is electrophysiological evidence for excitatory inputs from CA3 to the dentate granule cells [14]. There is no evidence on whether or not CA3 has any influence on the process of neurogenesis within the dentate gyrus, or on the ability of drugs such as fluoxetine to increase it. Should this occur, it would add a new dimension to our knowledge of the way that neurogenesis is controlled from within the hippocampus. It might also suggest additional sites of action for systemic agents, such as glucocorticoids, that control neurogenesis [8].

In this paper, we report the effects of small, highly-localised, unilateral lesions of CA3 on the basal levels of mitosis in the progenitor cells lining the innermost region of the granule cell layer of the dentate gyrus, on the survival of newly-formed neurons, and whether these lesions alter the mitotic response of progenitor cells to the SSRI fluoxetine. We also explore the effects these lesions have on the expression of BDNF (Brain derived neurotrophic factor), pCREB (Phosphorylated cyclic AMP response element) and Wnt3a, all known to be concerned in the regulation of progenitor cell mitosis [15], and on markers of synapse formation (GAP-43) and neuronal maturation (NeuN, calbindin) in the dentate gyrus [16], [17].

## Results

**Figure 1:**
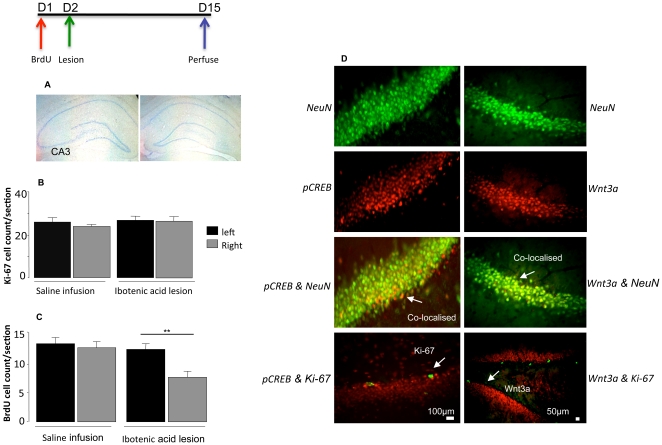
(A) Cresyl violet staining of the CA3 lesion following ibotenic acid infusions. (B) The effect of CA3 lesion on the newly formed neurons BrdU and the mitosis rates Ki-67 (C) of progenitor cells in the dentate gyrus. Mean number per section after 14 days, compared to controls (saline infusion). Values are mean ±SEM. **p<0.01, compared to control. (D) Immunofluorescent Co-localisation of NeuN stained in green, pCREB (left) and Wnt3a stained in red (right) in saline treated animals. Double labelling for NeuN/pCREB and NeuN/Wnt3a appears orange, Ki67 staining is green. Bar represents a distance of 100 µm and 50 µm.

Lesions were sharply restricted to CA3 area (Figure 1A) and did not encroach into the neighbouring dentate gyrus. Within the area of the lesion, there was virtually complete loss of pyramidal cells. Saline infusions had no discernible effect.

### Experiment 1. The effect of unilateral CA3 lesions on rates of progenitor proliferation

In the first experiment we asked whether a unilateral lesions of CA3 would alter the number of mitotic progenitor cells in the dentate gyrus after four days. There were no differences between lesioned and saline-infused animals in either the number of BrdU-positive cells (labelled for four days immediately before the lesion) (repeated measures ANOVA: F = 0.14; p:ns (not significant)) or Ki67-positive ones (mitotic at the time of sampling, four days after the lesion) (F = 6.4, p:ns). The number of BrdU positive cells in the control animals was 22.7±3.5 (mean ± SEM) on the right (saline infused) side compared to 21.7±1.2 on the left side, and in lesioned animals was 21.1±1.5 on the right (ibotenic acid) ) on the left (non-lesion) 20.8±1.6. Ki-67 counts were: in the control animals 20.2±0.8 (right) and 21.7+1.34 (left); for lesioned animals: 22.6 +1.1 and 20.8 +1.07 (left).

### Experiment 2: Effect of CA3 lesions on early-stage survival of newly-formed neurons in the dentate gyrus

We next examined whether lesions altered the survival of newly-formed neurons. In this case, there was still no difference in the number of Ki-67 labelled cells 15 days after a unilateral CA3 lesion (right side; Fig. 1A) compared to the left side (saline infusion) (F = 0.3, p:ns) (Figure 1B). However, there was a highly significant reduction in the number of BrdU-labelled cells on the lesioned (right) side (F = 6.4, p = 0.03) (Figure 1C), suggesting decreased survival of progenitor cells that were mitotic at the time of the lesion, and thus forming new neurons.

We wanted to determine whether pCREB or Wnt3a co-localised with either mature neurons or progenitor cells. Double-staining showed that Ki-67 did not co-localise with either pCREB or Wnt3a. However, both co-localised with NeuN, a marker of mature neurons (Figure 1D).

### Experiment 3. Effect of CA3 lesions on survival of new neurons after 28 days

**Figure 2:**
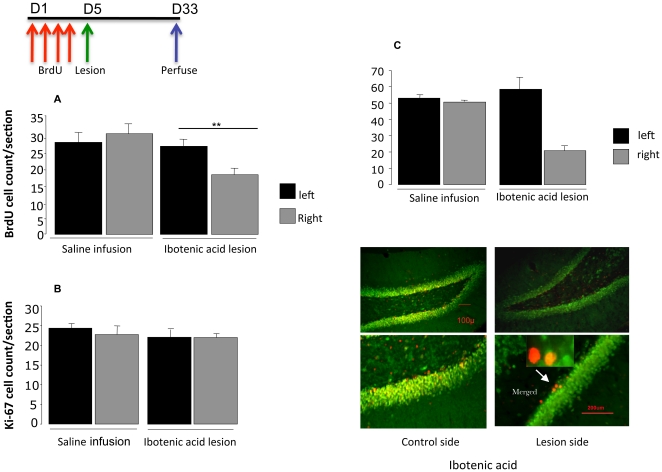
The effect of CA3 lesion on (A) the newly formed neurons staining for BrdU and (B) the number of Ki-67-positive progenitor cells in the dentate gyrus. Mean number per section after 28 days, compared to controls (saline infusion). Values are means ± SEM. Values are mean ±SEM. **p<0.01, p:ns compared to control. (C) double-stained cells with BrdU and NeuN. Bar represents a distance of 200 µm and 100 µm.

Since newly-formed neurons take around 28 days to mature, the next experimentexamined rats given pre-lesion BrdU and sampled after this interval. Pairwise comparison between infused (right) and non-infused (left) sides showed that there were fewer BrdU-stained cells on the ibotenic acid-infused animals (paired t-test: t = 5.5, p = 0.03) but not in saline-infused animals (t = 1.9, p:ns) (Figure 2A). There was no change in those labelled with Ki-67 (F = 0.2, p:ns) (Figure 2B). A repeated measures ANOVA including both time points showed a highly significant effect of lesioned vs non-lesioned side (F = 7.6, p = 0.012) but no effect of time (15 or 28 days) for BrdU labelling (p:ns). This was confirmed by double-staining cells with BrdU and NeuN, which showed that it was these cells that had decreased (F = 5.6, p = 0.05 (Figure 2C). There was no change in the expression of pCREB or Wnt3a in both experiment 2 and 3 (Table 1.)

**Table 1:** Mean Optical Density (pixels) for pCREB or Wnt3a after either saline or ibotenic acid lesions for 14 days (Experiment 2) or 28 days (Experiment 3) and following treatment with subcutaneous saline or fluoxetine-containing pumps for 14 (Experiment 4), 3 animals/group and 3 sections/animal.

TT	Wnt3a Mean OD ±SE Left	Wnt3a Mean OD ±SE Right (lesion)	pCREB Mean OD ±SE Left	pCREB Mean OD ±SE Right (lesion)
**Experiment 2**				
Saline lesion (14 days)	24.20±0.55	26.04±0.21	29.97±0.05	29.00±2.1
Ibotenic acid lesion (14 days)	26.19±1.55	27.16±2.54	29.33±0.88	29.35±0.69
**Experiment 3**				
Saline lesion (28 days)	27.09±0.73	27.28±0.53	26.15±1.11	26.48±0.63
Ibotenic acid lesion (28 days6	26.59±0.75	27.51±1.33	26.11±1.20	26.30±1.47
**Experiment 4**				
Saline pumps (14 days)	28.00±1.85	27.24±1.39	28.99±0.89	27.62±0.44
Fluoxetine pumps 10 mg/kg/day (14 days)	87.64±1.57	87.21±1.42	97.45±1.50	98.43±0.45

### Experiment 4. Effect of CA3 lesions on the ability of fluoxetine to stimulate progenitor cell mitosis in the dentate gyrus

Both previous experiments had shown that basal levels of mitosis in the progenitor cells were not altered by CA3 lesions, although subsequent survival of these cells were highly compromised. There is evidence that the requirements for increasing mitosis may differ from basal conditions. For example, blocking trkB receptors has no effect on basal rates, but prevents fluoxetine from increasing them [15]. So we asked whether fluoxetine retained its ability to stimulate progenitor mitosis.

**Figure 3:**
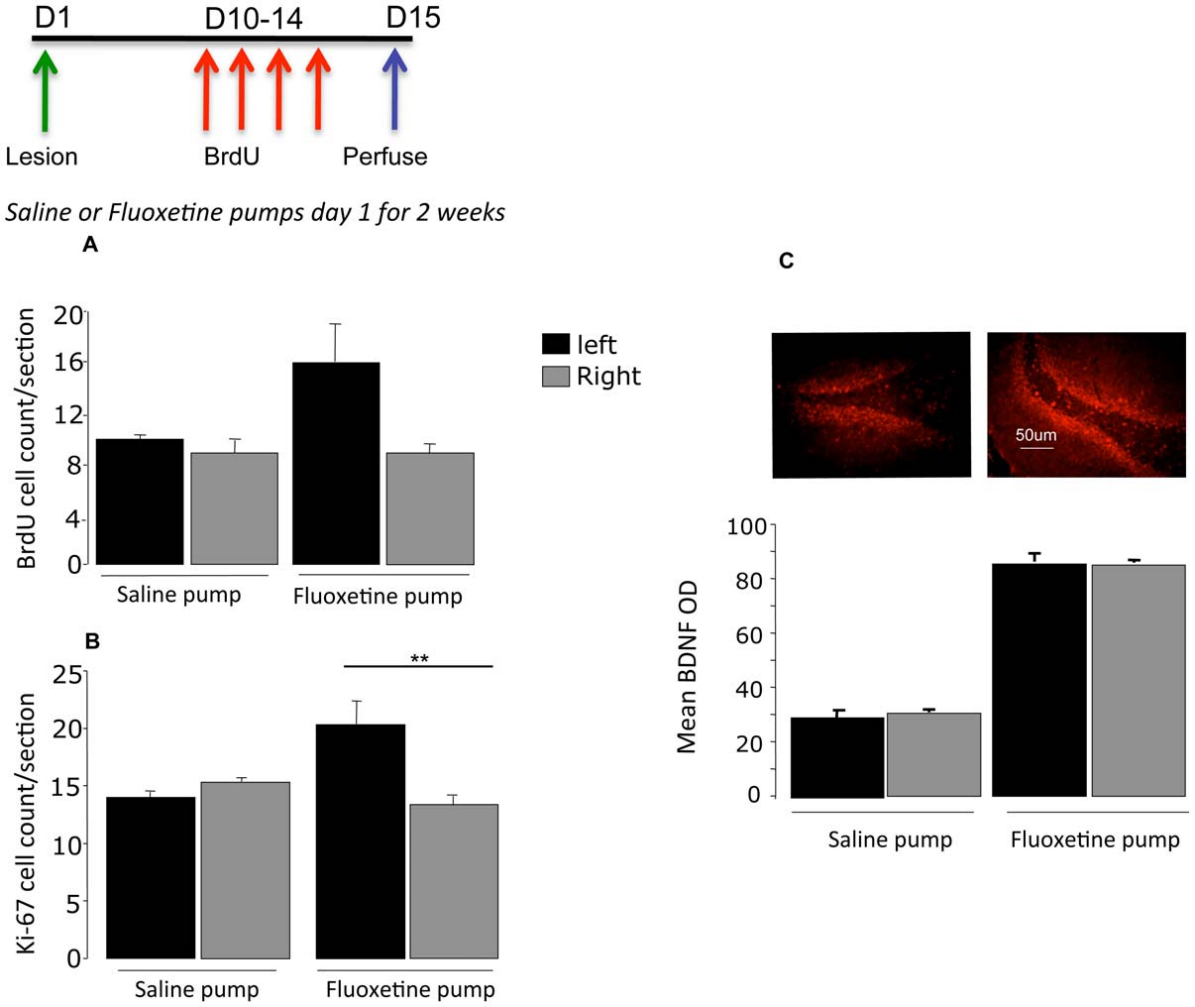
The effect of CA3 lesions on (A) the survival of BrdU cells and (B) number of Ki-67+ progenitor cells in the dentate gyrus after 14 days treatment with either fluoxetine (10 mg/kg/day) or saline subcutaneously (osmotic minipump). Values are means ± SEM., **p<0.01 compared to control. (C) Immunofluorescent staining of BDNF after the two treatments. Values are mean optical density of BDNF ±SEM. **p<0.001. Bar represents a distance of 50 µm.

Rats with unilateral CA3 lesions and implanted with osmotic minipumps releasing fluoxetine (10 mg/kg/day) failed to show the expected increase in Ki-67 labelled cells on the lesioned side after 14 days treatment, compared to the unlesioned side (F = 11.7, p = 0.009) (Figure 3B). Although there seemed to be a similar decrease in BrdU-labelled cells on the lesioned side in fluoxetine-treated animals, this was not significant (F = 2.7, p:ns)(Figure 3A)

Since fluoxetine no longer stimulated progenitor mitosis after a CA3 lesion, we wanted to know whether we could determine more precisely how this happened. It is known that BDNF is increased by fluoxetine [18], and that this seems essential for increased progenitor mitosis [15]. Double-staining showed that a number of NeuN-expressing cells also stained for BDNF. CA3 lesions themselves had no effect on BDNF in the dentate gyrus after 14 days in experiment 4. Fluoxetine, as expected, increased BDNF expression following 14 days fluoxetine treatment in both control-lesioned rats and on the unlesioned side in those with a CA3 lesion (F = 23.2, p<0.001). However, it also increased BDNF on the lesioned side, and there was no difference between the two sides (F = 0.67, p:ns) (Figure 3C).

Since increases in both pCREB and Wnt3a expression in the dentate gyrus accompany fluoxetine-stimulated progenitor mitosis, we next asked whether this still occurred after a CA3 lesion As expected, fluoxetine treatment for 14 days increased the expression of both in control rats and on the unlesioned side (pCREB, p<0.001, Wnt3a p<0.001) but there was no difference between the lesioned and non-lesioned sides (side x lesion; p:ns in both cases) (Table 1).

**Figure 4:**
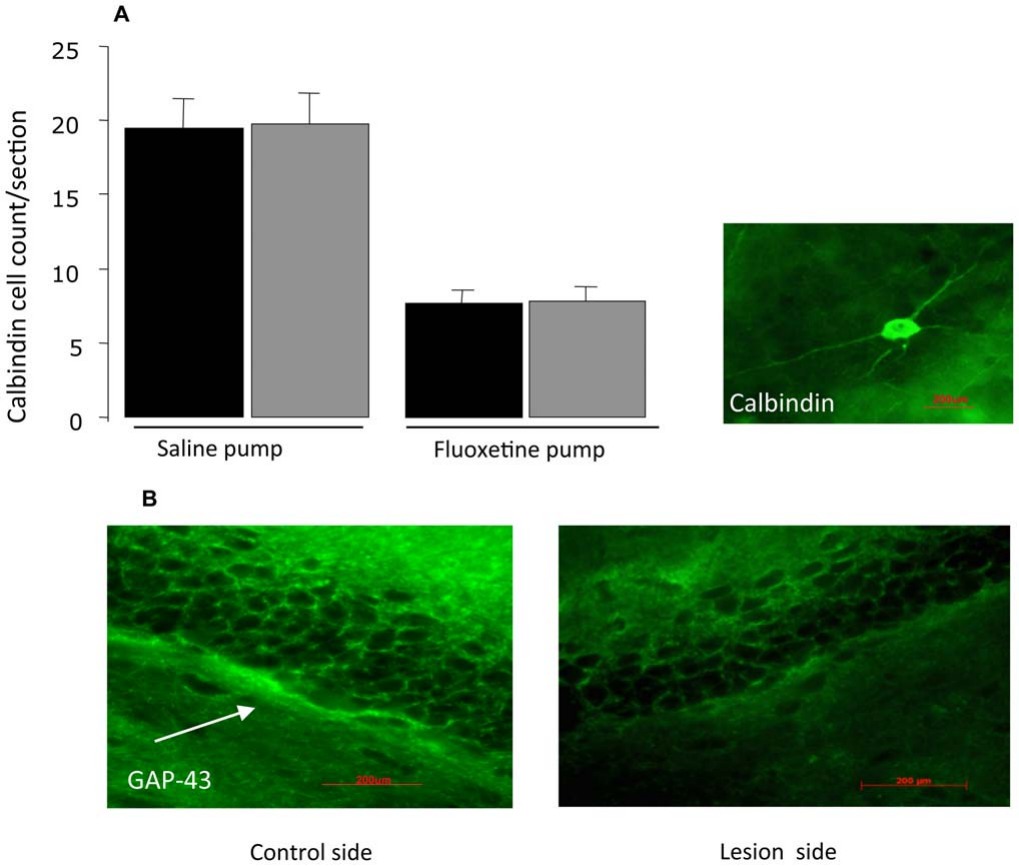
The number of calbindin-positive cells in the dentate gyrus after. (A) CA3 lesiona combined with either fluoxetine (10 mg/kg/day) or saline subcutaneously (minipumps for 14 days). (B) Immunofluorescent staining of GAP-43 on the lesion edside compared to the non lesioned side. Bar represents a distance of 200 µm.

Fluoxetine has been reported to decrease the maturation of newly-formed neurons as defined by their expression of calbindin [17]. We confirmed that, on the non-lesioned side, fluoxetine reduced the number of calbindin-positive cells (F = 160, p<0.001). However, this still occurred on the CA3-lesioned side and there was no difference between them (F = 0.14, p:ns) (Figure 4A).

Since there is evidence for projections from CA3 to the dentate gyrus (see Introduction) we examined the effect of a CA3 lesion on expression of GAP-43 in the dentate gyrus. There was a marked reduction by visual inspection of GAP-43 within the granule cells on the side of a CA3 lesion compared to the non-lesioned side (Figure 4B).

## Discussion

This paper shows that lesions of the hippocampal CA3 have major effects on neurogenesis in the dentate gyrus. This has never been reported before. Since we have not explored the effects of lesions in other CA fields, we cannot be sure at this stage that these results are specific to CA3, though, because of the particular relation between this field and the dentate gyrus, it seems likely that they will prove to be so. Our first finding was that basal levels of progenitor cell mitosis were not altered by a CA3 lesion, but the dentate gyrus was seemingly unable to respond to a well-established treatment - fluoxetine - that increases it [4]. Since effects on basal or stimulated rates of mitosis may differ (eg a flattened corticoid rhythm: [4]), it is important to define ‘basal’ levels. This is always problematic, particularly in the case of hippocampal neurogenesis which is so sensitive to a variety of environmental influences (see Introduction). In particular, perturbations of adrenocortical activity can easily alter rates of neurogenesis [19], [20], [21]. However, the rats we used were well-accustomed to their standardised housing and social environment, so could be credibly classified as being in a ‘basal’ state. Both our first experiment, in which we measured Ki-67-labelled cells, and the fourth one, where we gave BrdU up to 24 hours before sacrifice, confirmed that CA3 lesions had no effect on basal levels of progenitor mitosis.

However, we found that fluoxetine was no longer able to increase the mitotoic rate of progenitor cells in the dentate gyrus in the presence of a CA3 lesion. Reports on the ability of this drug to stimulate mitosis have differed. Some confirm a positive result [22], [23] but others do not [24], [25]. Emerging evidence suggests that route of administration (oral, subcutaneous injection, osmotic minipump) is important: only minipump infusions have reliably increased mitosis rates. The solvent used is another factor: propylene glycol itself increases mitosis rates (our unpublised data).

Our finding that there are differential effects of a treatment (fluoxetine) on stimulated progenitor cell mitosis, but not basal levels, echoes previous reports. We find, for example, that blocking trkB receptors with the drug K252a has no effect on basal mitosis rates, but prevents the increase which otherwise follows treatment with fluoxetine [15]. This suggests that particular mechanisms are involved in raising mitosis rates in response to modifications in serotonin activity. This is not because basal levels cannot be reduced: treatment with excess corticoids suppresses mitosis to well below the basal rate [26]. Previous results have also shown that BDNF is involved in the response to progenitor cell to fluoxetine [27], [28]. We therefore sought to determine whether the expected increase in BDNF content in the dentate gyrus following fluoxetine treatment might have been prevented by CA3 lesions. But this was not the case. The anticipated increase in BDNF following changed serotonin still occurred. Since intracerebroventricular infusions of BDNF increase progenitor mitosis in the dentate gyrus [29], one question raised by this result was why this did not occur despite raised BDNF.

We therefore examined the expression of pCREB and Wnt3a. We have previously shown that both increase in the dentate gyrus after fluoxetine treatment, together with BDNF [15]. However, the canonical intracellular pathway responding to Wnt3a (β-catenin) is distinct from that for BDNF, which operates via pCREB - though the relationship between BDNF and pCREB is two-way and complex [30]). It was possible that either or both of these intracellular components might have been affected by CA3 lesions, and thus could explain why fluoxetine no longer activated progenitor cell mitosis. But we found that the expected increase in both pCREB and Wnt3a expression still occurred in the dentate gyrus following fluoxetine treatment on the side of a CA3 lesion, even though no change in progenitor cell mitosis from basal levels was observed. To test whether another known effect of fluoxetine was sensitive to this lesion, we examined the expression of calbindin. It has been reported that fluoxetine can reduce the expression of calbindin in the dentate gyrus, an effect which is held to be evidence for a ‘de-maturation’ of newly-formed neurons, since calbindin is typically expressed by mature ones [17]. We confirmed that rats treated with fluoxetine showed reduced levels of calbindin expression; but this also occurred on the side of a CA3 lesion. It appear that the inhibitory effects of a CA3 lesion on the efficacy of fluoxetine (and hence, it would seem, serotonin) is not a generalised one, and does not necessarily apply to all its actions on the dentate gyrus. However, our findings do show that activation of a range of cellular events known to be associated with, and possibly responsible for, accelerating the rate of mitosis on the progenitor cells of the dentate gyrus still occur after a CA3 lesion; nevertheless, the expected increase no longer occurs.

In order to demonstrate directly a connection between CA3 and the dentate gyrus we stained the latter for GAP-43, a synaptic marker [31]. We found that GAP-43 was depleted in lesioned animals. This supports the evidence already cited of a connection between the two structures, though ascribing these synapses to a projection from CA3 is only a reasonable assumption. Electrophysiological recordings have shown that this connection is largely excitatory to the basket cells in the dentate gyrus, which themselves inhibit the granule cells, and hence mossy fibre output back to CA3 [14]. We did not specifically examine mossy fibres within the dentate gyrus, but there are reports that CA3 lesions induce aberrant sprouting of these fibres [12], [32]. Whether either of these events is responsible for the findings reported here remains an open question. However, since the expected alterations in BDNF, pCREB and Wnt3a still occurred in CA3-lesioned animals, it does seem that this serotonin-acting drug acts on the dentate gyrus itself, rather than primarily on CA3, which may have a permissive role. Whether this applies to other regulatory agents, such as glucocorticoids, is still not determined.

Our finding that there is a marked decrease in survival of newly-born neurons after CA3 lesions is more easily explained. Since these cells ordinarily project to CA3, the loss of this terminal area might be expected to reduce their ability to make connections and thus survive. Survival even under normal conditions is only around 50% [33], so it is not surprising that removal of their target area further compromises these cells. Our results suggest that one mechanism for the failure of so many new neurons to survive even under basal conditions may be competition for efferent contact with receptor neurons in CA3.

The results reported here open a new window on the control of neurogenesis in the adult hippocampus. They suggest that regulation does not reside entirely within the dentate gyrus, the site of neurogenesis, but that the projection area of newly-formed neurons, CA3, exerts a hitherto unknown but potent reciprocal influence over the activation of neurogenesis in the adult brain at least by one class of anti-depressants. Whether this applies to others is still to be determined.

## Materials and Methods

### Animals

All procedures were carried out under Home Office (UK) licence (PPL80/1966). Male Sprague-Dawley rats (Harlan, Oxon, UK) were used, weighing 200–250 grams at the start of the experiment. Rats were housed individually in a controlled environment. Ambient temperature was maintained at 21°C and humidity at 55% with *ad* libitum access to food and tap wate*r*. Animals were kept in a reversed 12-h light: 12-h dark cycle (lights off at 10.00 hours).

### Experimental design

#### Experiment 1

In the first experiment we asked whether a unilateral lesions of CA3 would alter the mitosis rates of the progenitor cells in the dentate gyrus in the short term. Two groups of rats (n = 3 per group) received four BrdU injection 50 mg/kg/day intraperitoneally for four days. On day 5 a unilateral lesion (ibotenic acid, see above) was made or an equivalent volume of saline was infused into CA3. The animals were killed on day 9.

#### Experiment 2

The next experiment tested whether CA3 lesions altered the survival of early-stage newly-formed neurons. Animals (n = 6 per group) received a single BrdU injection (200 mg/kg) the day before either CA3 lesions or control infusions were made as in the previous experiment. However, they were killed 15 days after the lesion.

#### Experiment 3

Since progenitor cells take around 28 days to fully mature, the third experiment examined rats which had been given BrdU (50 mg/kg/day) for 4 days, then received either CA3 lesions or control infusions unilaterally as above, and sampled after 28 days. (n = 6 per group)

#### Experiment 4

This examined the ability of progenitor cells in rats with CA3 lesions to respond to fluoxetine (Fluoxetine hydrochloride:Sigma Dorset,UK, catalogue number F-132). Rats (n = 5 per group) received CA3 lesions on day1, and were also implanted with subcutaneous osmotic minipumps in the posterior upper thorax (model 2ML2 the rate of flow is 5 µl/hr for 14 days) (Charles River, Margate, UK, catalogue number 2ML2) filled either with fluoxetine (releasing 10 mg/kg/day dissolved directly in saline under running hot tap water or saline, therefore for animals weighing 250 gm they would receive 46.6 mg of fluoxetine in one osmotic minipump,. They also received four sc injections of BrdU (50 mg/kg/day) for four days starting on day 10. They were killed 14 days later.

### Ibotenic acid lesions

Rats were anesthetised using isofluorane, oxygen and NO and placed securely into a stereotaxic frame (David Kopt instruments,Tujunga,CA,USA). The dorsal surface of the skull was exposed, a hole was drilled and a cannula inserted A 5 µl Hamilton syringe fitted with a 30-gauge cannula and filled with ibotenic acid solution (Sigma,Dorset,UK,catalogue number 12765) (10 mg/ml;0.2 µl/site) was inserted using the following coordinates: 2.7–4.7 mm caudal to bregma (AP), 4.1 mm right from the mid line (L) and 4.5 mm from the cortical surface (V) ([34]. Infusions were made at three AP sites 1 mm apart. The flow rate was 0.6 µl/3 min for each site using an auto injector device (Cole-Palmer, London UK). The cannula was left in place for 2 min and slowly retracted thereafter. Control rats were subjected to the same procedure except that saline was injected.

### Brain fixation and histology

Rats were deeply anesthetised with a terminal dose of pentobarbital sodium and perfused transcardially with 0.1 M phosphate-buffered saline (PBS, pH 7.4), followed by 4% paraformaldehyde in PB. Brains were removed and post-fixed in the same fixative for 4 hours then transferred to 30% sucrose in PBS overnight.

Serial coronal sections (40 µm) were cut with a freezing microtome through the entire length of the dorsal hippocampus (from−2.80 to −4.52 mm behind bregma), placed into a well of anti-freeze (1∶1∶2 glycerol:ethylene glycol: 0.1 PBS) and kept at −20°C until use.

### Quantification

#### Proliferating cells Ki-67 and BrdU

Slides were randomised and coded prior to quantitative analysis. Labelled cells on each side of the brain were counted using a 40X objective; only cells on the internal border of the subgranular zone of the dentate gyrus were included. The data shown are the mean number of Ki-67 or BrdU labelled cells per section from 12 sections (1 in 6) per animal; starting from 2.80 to −4.52 mm behind bregma.

#### Analysis of pCREB, Wnt3a, BDNF

Three sections per animal (1∶12 starting between bregma −3.14 mm and −3.30 mm) were used.;.After staining the images were captured (x10 objective) using AxioCam digital camera attached to Carl Zeiss microscope they were then displayed on a computer screen where AxioVision application software were used they were standardised by setting the exposure and brightness to a constant value The images were then saved in JPEG format, and analysed using Image J-64. On each section 3–4 circles (59.2 µm diameter) 200 µm apart were drawn on each blade of the dentate gyrus, starting 400 µm from the hilus, and the OD (optical density) measured in each. Results are given as mean OD per animal (Table 1).

***Calbindin:*** positive cells were counted on each side of the dentate gyrus (CA3 lesioned and non lesioned sides) on three sections per animal in Experiment 4.

### Statistical analysis

Within animal comparisons (between lesioned and non-lesioned sides) were analysed with repeated measures ANOVA. Between animal comparisons were made by multivariate ANOVA or t-tests, as appropriate. Homogeneity of variance was checked with Levene's test.

### Immunohistochemistry

#### Single Staining

***Ki-67*** **:** sections were incubated in 0.01 M citric acid for 40 min at 98°C. They were cooled and washed twice ×5 min in KPBS (Potassium phosphate buffer saline). Endogenous peroxidase activity was quenched with 3% H_2_O_2_ solution for 10 min followed two 5 min washes with KPBS. Then they were incubated with primary antibody (1∶100 mouse monoclonal IgG anti-human Ki-67; Novocastra, Newcastle Upon Tyne, UK, catalogue number VP-K452) and 1% horse serum in a humidified chamber at room temperature overnight. The next day, after two × 5 min washes with KPBS, they were incubated with secondary antibody (1∶200 biotinylated mouse IgG; Vector laboratories Ltd, Peterborough UK, catalogue number 6102) for 1 hour at room temperature. After two 5 min washes with KPBS, they were incubated with Avidin-Biotin-Peroxidase (Vector laboratories Ltd, Peterborough UK) for a further hour, followed by two 5 min washes with KPBS. The staining was visualized using DAB (3,3-diaminobenzidin) tablets (Sigma, Dorset, UK) for 5 min.

Slides were then counterstained with 10% cresyl violet solution followed by dehydration through ethanol and Histoclear. They were cover-slipped with DPX (Distyrene-xylene;BDH,Leicestershire,UK) for light microscopy.

***BrdU*** **:** sections were mounted on poly-lysin-coated slides (BDH, UK),dried overnight, incubated in 0.01 M citric acid for 15 min at 97°C, then in 3% H_2_O_2_ for 10 min, digested in trypsin (0.025%) for 10 min, denatured in 2N HCl for 30 min at 37° C, rinsed, and incubated in mouse monoclonal antibody raised against BrdU (Novocastra Newcastle Upon Tyne, UK, catalogue number VP-B209) 1∶200 in 0.3% triton,1% normal horse serum) overnight at room temperature. The slides were rinsed and taken through a mouse IgG ABC kit procedure (Vector), rinsed again and reacted for 5 min with DAB tablets (Sigma), rinsed, dehydrated and cover slipped under DPX as above.

#### Fluorescent Single & Double staining

***pCREB and Wnt3a single staining*** **:** Free floating sections from each animal (1 in 12) were incubated in rabbit anti-pCREB (Cell Signaling, Hitchen, UK, catalogue number 9198; 1∶25 in 0.3% Triton with 1% goat serum) overnight in a humidified chamber at room temperature. After two washes with KPBS, sections were incubated in Alexa Fluor 568 (Invitrogen, Paisley UK, catalogue number A-11011) goat anti-rabbit (1∶200 in 0.5% Triton) for one hour, washed twice with KPBS, and cover-slipped.

For staining with Wnt3a, sections were incubated in rabbit anti-Wnt3a (Abcam, Science Park, Cambridge UK, catalogue number ab-28472; 5 µg/ml in 0.3% Triton with 1% goat serum) overnight in a humidified chamber at room temperature. The following day, after two washes with KPBS, they were incubated in Alexa Fluor 568 goat anti-rabbit (1∶200 in 0.5% Triton) for one hour, and washed twice with KPBS.

***BDNF*** **:** sections were incubated in rabbit anti-BDNF (Santa Cruz Biotechnology, Wembley, Middlesex, UK, catalogue number Sc-20981; 1∶100 in 0.3% Triton with 1% goat serum) overnight in a humidified chamber at 4°C. After two washes with KPBS, the sections were incubated in Alexa Fluor 568 (Invitrogen, Paisley, UK) goat anti-rabbit (1∶500 in 0.3% Triton) for one hour, washed twice with KPBS, and cover-slipped.

***BrdU and NeuN double labelling*** **:** Free floating sections from each animal (1 in 12; between bregma −3.14 mm and −3.30 mm) were washed twice with KPBS for 5 min and then denatured in 2N HCl for 30 min at 37°C, rinsed and then incubated for double label in a mixture of mouse anti NeuN (Millipore, Chandlers Ford, Hants, UK, catalogue number MAB377) (1∶100 in [0.3% Triton in KPBS] with 1% goat serum) and rat anti-BrdU (Accurate Chemical &Scientific Corporation, Westbury,USA, catalogue number OBT0030) (1∶200 in 0.3% Triton with 1% goat serum) overnight at 4°C. The next day after two washes with KPBS, the sections were incubated in a mixture of Alexa Fluor 568 goat anti rat catalogue number A-11077 [red] (1∶200) and goat anti mouse Alexa Fluor 488 catalogue number A-11001 [green] 1∶200 in 0.3% Triton + 1% goat serum for one hour, washed twice with KPBS, mounted on slides and cover slipped using a mixture of 50% glycerol and KPBS. Co-localized cells were coloured orange.

***Wnt3a and NeuN or pCREB and NeuN*** **:** Free floating sections were incubated overnight at 4°C in a mixture of rabbit anti Wnt3a (Abcam, Science Park, Cambridge UK; 5 µg/ml in 0.3% Triton with 1% goat serum) and NeuN (Millipore, Chandlers Ford, Hants, UK) (1∶00 in [0.3% Triton in KPBS] with 1% goat serum) or a mixture of rabbit anti pCREB and NeuN as above. The next day after two washes with KPBS, the sections were incubated in a mixture of Alexa Fluor 568 goat anti rabbit [red] (1∶200) and goat anti mouse Alexa Fluor 488 [green] 1∶200 in 0.3% Triton+1% goat serum for one hour, washed twice with KPBS, mount the sections on slide and cover slipped using a mixture of 50% glycerol and KPBS. Co-localized cells were orange.

***GAP-43:*** Free floating sections were incubated with mouse anti GAP-43 (Millipore, Chandlers Ford, Hants, UK, catalogue number MAB347 1∶400 in 0.3% Triton in KPBS with 1% goat serum overnight at 4°C. The next day, after two washes with KPBS, the sections were incubated in goat anti mouse Alexa Fluor 488[green] 1∶200 in 0.3% Triton+1% goat serum for one hour, washed twice with KPBS, mount the sections on slide and cover slipped using a mixture of 50% glycerol and KPBS.

#### Calbindin

Free floating sections were incubated with mouse anti Calbindin (Swant, Bellinzona, Switzerland, catalogue number 300) 1∶5000 in 0.4% Triton in KPBS with 1% goat serum overnight at 4°C. The next day, after two washes with KPBS, the sections were incubated in goat anti mouse Alexa Fluor 488[green] 1∶500 in 0.4% Triton+1% goat serum for one hour, washed twice with KPBS, mount the sections on slide and cover slipped using a mixture of 50% glycerol and KPBS.
